# Individual target pharmacokinetic/pharmacodynamic attainment rates among cefepime-treated patients admitted to the ICU with hospital-acquired pneumonia with and without ECMO

**DOI:** 10.1128/aac.00102-25

**Published:** 2025-05-15

**Authors:** Adrian Valadez, Marta Zurawska, Emma Harlan, Marc H. Scheetz, MIchael N. Neely, Paul R. Yarnold, Mengjia Kang, Erin Korth, Francisco Martinez, Bridget Giblin, Helen K. Donnelly, Kay Dedicatoria, Rachel Medernach, Sophia Nozick, Alan R. Hauser, Egon A. Ozer, Estefani Diaz, Alexander V. Misharin, Richard G. Wunderink, Nathaniel J. Rhodes

**Affiliations:** 1 Department of Pharmacy Practice, College of Pharmacy, Midwestern University, Downers Grove, Illinois, USA; 2 Pharmacometrics Center of Excellence, Midwestern University, Downers Grove, Illinois, USA; 3 Department of Pharmacy, Northwestern Memorial Hospital, Chicago, Illinois, USA; 4 Department of Pharmacology and Biomedical Sciences, College of Graduate Studies, Midwestern University 24560, Downers Grove, Illinois, USA; 5 Keck School of Medicine, University of Southern California 5116, Los Angeles, California, USA; 6 Laboratory of Applied Pharmacokinetics and Bioinformatics, The Saban Research Institute, Children's Hospital of Los Angeles, Los Angeles, California, USA; 7 Optimal Data Analysis, LLC, Chicago, Illinois, USA; 8 Division of Pulmonary and Critical Care Medicine, Department of Medicine, Northwestern University Feinberg School of Medicine 12244, Chicago, Illinois, USA; 9 Division of Infectious Diseases, Department of Internal Medicine, RUSH University Medical Center 726754, Chicago, Illinois, USA; 10 Department of Microbiology-Immunology, Northwestern University Feinberg School of Medicine 12244, Chicago, Illinois, USA; 11 Division of Infectious Diseases, Department of Medicine, Northwestern University Feinberg School of Medicine 12244, Chicago, Illinois, USA; 12 Center for Pathogen Genomics and Microbial Evolution, Havey Institute for Global Health, Feinberg School of Medicine, Northwestern University 12244, Chicago, Illinois, USA; 13 Robert H. Lurie Comprehensive Cancer Research Center, Feinberg School of Medicine, Northwestern University 12244, Chicago, Illinois, USA; Providence Portland Medical Center, Portland, Oregon, USA

**Keywords:** pharmacokinetics extracorporeal membrane oxygenation

## Abstract

Cefepime (FEP) is used for hospital- and ventilator-associated pneumonia when *Pseudomonas aeruginosa* is involved. However, its pharmacokinetics (PK) in severe pneumonia necessitating extracorporeal membrane oxygenation (ECMO) remain unclear. This single-center, prospective study enrolled 70 mechanically ventilated patients with suspected pneumonia (*n* = 9 on ECMO), excluding those on renal replacement therapy. Dosing followed institutional renal function-based protocols. Plasma concentrations were quantified by liquid chromatography-tandem mass spectrometry, and a two-compartment PK model was developed using *Pmetrics* for R, with volume of distribution (Vd) scaled to body weight and ECMO status, and clearance (CL) scaled to renal function. Target attainment was calculated from Bayesian posterior predictions, and Monte Carlo simulations evaluated the cumulative fraction of response (CFR) for regimens of 2 g IV every 8 h, administered as either 0.5 h intermittent or 4 h extended infusion with or without a 2 or 3 g loading dose (LD) (0.5 h). Success was defined as achieving 100% *f*T _>1xMIC_ within 24 h for 80% of isolates. Seventy patients (60% male, *n* = 9 ECMO) contributed 114 plasma samples (1–14 per patient). The model fit the data well. ECMO was associated with a 2.8-fold increase in Vd without altering CL. Monte Carlo simulations demonstrated that standard dosing without an LD failed to achieve CFR ≥ 80% in ECMO patients. Incorporating a 3 g but not 2 g LD restored CFR to ≥80% in ECMO. ECMO significantly increased FEP Vd in intensive care unit patients, suggesting sub-optimal target attainment at higher minimum inhibitory concentrations. A 3 g LD appears essential for target attainment, underscoring the need for revised dosing strategies in ECMO.

## INTRODUCTION

Hospital-acquired pneumonia is a significant cause of infection in the intensive care unit (1, 2), with mortality rates ranging from 20% to 50% (3–5). Broad-spectrum antibiotics, such as cefepime, are often required to empirically treat pathogens that cause invasive infections, such as *Pseudomonas aeruginosa* and Enterobacterales (6). Due to pathophysiological changes in critically ill patients requiring extracorporeal membrane oxygenation (ECMO), the pharmacokinetics (PK) of beta-lactams can be altered, particularly through expanded volume of distribution (Vd) (7). ECMO is a form of life support used in patients with severe respiratory or cardiac failure, in which blood is oxygenated outside the body to relieve the workload on the heart and lungs. ECMO is increasingly used in patients with severe respiratory failure, but the effect of the ECMO circuit on cefepime PK parameters is not well understood. Although *ex vivo* studies suggest minimal sequestration of cefepime by ECMO circuits (8, 9), clinical evidence highlights variability in cefepime exposures (10–12). This variability likely reflects cefepime's hydrophilic nature and its characteristic low Vd and low logP, which describe the drug’s tendency to remain in aqueous compartments rather than fat or tissue (13). In the context of ECMO, the expanded extracellular fluid volume in critically ill patients may lead to an increased Vd, potentially resulting in suboptimal target attainment. However, prior studies of the effect of ECMO on cefepime PK included patients on concurrent renal replacement therapy (RRT), complicating the analysis of ECMO-specific effects on PK.

Guidelines for beta-lactam use in critically ill patients suggest 100% *f*T_>1xMIC_ as a minimum pharmacokinetic-pharmacodynamic (PK/PD) target to improve the probability of clinical response (14). However, 100% *f*T_>4xMIC_ has also been suggested as a more aggressive target in the critically ill population, as this target has also been associated with improved clinical outcomes (15, 16). Given the potential benefits to critically ill patients of increasing *f*T_>MIC_ (15), identifying clinical factors (e.g., ECMO) which can alter PK/PD target attainment is important for optimal dosing strategies. However, excessive cefepime exposure has been associated with neurotoxicity, reinforcing the need for careful dose optimization. Recent studies have highlighted the risk of cefepime neurotoxicity, particularly at higher trough concentrations, with patients exhibiting renal dysfunction being at greater risk (17). Furthermore, careful monitoring of cefepime concentrations may help mitigate the risk of neurotoxic effects while ensuring therapeutic efficacy (18). We hypothesized that ECMO increases Vd but only minimally affects clearance (CL), leading to reduced *f*T_>MIC_ in plasma. In this study, we evaluate patient-specific and population PK/PD attainment in critically ill patients treated with cefepime, with and without ECMO, and not requiring RRT.

## MATERIALS AND METHODS

### Design and patients

This was a retrospective PK/PD study nested within the Successful Clinical Response in Pneumonia Treatment (SCRIPT) study (19). Residual plasma samples were collected from patients admitted to the Medical Intensive Care Unit at Northwestern Memorial Hospital (Chicago, IL, USA) with suspected pneumonia between June 2018 and March 2024. Patients with and without ECMO were included, while those requiring concurrent hemodialysis (HD), peritoneal dialysis, or continuous renal replacement therapy (CRRT) were excluded. Covariate data, including demographic (age, sex), morphometric (weight, body surface area [BSA]), and clinical (serum creatinine, ECMO status, and dosing records) parameters, were extracted from electronic health record (EPIC, Verona, WI) and aggregated using the Northwestern Enterprise Data Warehouse. Creatinine clearance (CrCl) was calculated using the method of Cockcroft and Gault equation (20).

### Cefepime dosing, sample collection, and bioanalysis

Cefepime dosing followed institutional protocols tailored to patients’ renal function and suspected pneumonia diagnosis (21). Residual blood samples were collected opportunistically and processed per protocol. Residual blood samples collected in lithium heparin tubes (BD Vacutainer) were held at 4°C for up to 48 h after the initial blood collection (22, 23). Plasma separation was performed within the clinical laboratory at Northwestern Memorial Hospital upon sample receipt. Samples were aliquoted and stored at −80°C until PK analysis.

Cefepime plasma concentrations were quantified using a validated liquid chromatography-tandem mass spectrometry (LC-MS/MS) assay. Total cefepime concentrations were quantified in plasma after protein precipitation with LC-MS-grade methanol (Sigma Aldrich, St. Louis, MO) and 0.1% formic acid (VWR Chemicals, Chicago, IL). Samples were analyzed using the Agilent 1260 Infinity II LC coupled to the Ultivo Triple Quadrupole MS/MS (Agilent Technologies, Santa Clara, CA). Analytical separation was achieved using a reverse-phase Kinetix Polar C18 column (50 mm × 2.1 mm × 2.6 µm; Phenomenex, Torrence, CA). Mobile phases A and B were 0.1% formic acid in deionized water and LC-MS-grade acetonitrile (VWR Chemicals, Chicago, IL) respectively. The mass transitions (m/z) for cefepime calibrators (MedChemExpress, Monmouth Junction, NJ) included 241.1 → 84 for quantification and 241.1 → 85 for qualification, with cefepime-d3 (m/z 242.6 → 87) as an internal standard (LCG, Manchester, NH). The assay was linear from 1 to 100 mg/L (*R*^2^ = 0.999). Accuracy (inter-day: 99.2%–101.9%; intra-day: 97.9%–102.8%) and precision (inter-day coefficient of variation [CV%]: 0.3%–1.7%; intra-day CV%: 0.8%–1.1%) met FDA requirements for bioanalytical method validation (24).

### Model development

Pharmacokinetic modeling was performed using the NPAG algorithm (25) available within the Pmetrics package (version 2.1.1) (26) for R (version 4.1.2) (27). Multiple compartmental models were considered. One-compartment and two-compartment models were evaluated. Covariate effects were evaluated in subsequent models wherein a significant change in model fitness was defined as an objective function value change between nested models (ΔOFV) of 3.84 with forward addition and backward elimination. Given our study hypothesis, the effect of ECMO on CL and Vd was evaluated *a priori*. Improvements in goodness of fit, log-likelihood, bias/imprecision, and Akaike Information Criterion (AIC) were used to select the final model.

### Covariate model build

We evaluated the effect of patient-specific covariates using regressions of the observed covariate vs PK parameter relationships for continuous variables (e.g., CrCl and total body weight) or boxplots for categorical variables (e.g., sex and ECMO status). Both linear and non-linear relationships between PK parameters and continuous covariates were evaluated.

### Individual PK/PD target attainment

Individual PK/PD target attainment rates were evaluated from patient data using Bayesian posterior predictions (23, 28). For each patient, we calculated the percentage of the dosing interval during which unbound cefepime concentrations remained above the MIC over the first 24 h of treatment (*f*T_>MIC_). Free drug concentrations were calculated applying the published protein binding of 20% (i.e., 1-protein binding) (21). Two PK/PD targets were assessed: 100% *f*T_>1xMIC_ and 100% *f*T_>4xMIC_, based on guidance for critically ill patients (14). These targets were applied to the CLSI breakpoint MICs of Enterobacterales (2 mg/L) and *P. aeruginosa* (8 mg/L) (29). Results were stratified by ECMO status.

### Monte Carlo simulations

While individual PK/PD target attainment was evaluated using actual patient data and Bayesian posterior predictions, we used Monte Carlo simulations to assess the probability of achieving target exposures across a range of dosing regimens and MIC distributions, providing additional insight into dosing strategies under varying clinical conditions. Monte Carlo simulations were conducted using the final covariate-adjusted compartmental PK model using the Pmetrics package for R (26). Simulations assessed the probability of target attainment (PTA) at fixed MICs of 2 and 8 mg/L as well as the cumulative fraction of response (CFR) vs the EUCAST MIC distribution (Fig. S1) for *P. aeruginosa* (30) as a worst-case scenario. In all simulations, we evaluated PTA and CFR against a PK/PD target of 100% *f*T_>1xMIC_. Renal clearance was held constant at the overall sample mean (CrCl = 115 mL/min) to focus on the effect of concurrent ECMO in the absence of renal impairment. We evaluated the following regimens: 2 g IV every 8 h, both with and without a 2 g loading dose (LD). When given, the LD was always administered as 2 g over 30 min, immediately followed by the maintenance regimen, which was either infused over 4 h (extended infusion, EI) or 0.5 h (intermittent infusion, II). To further assess the impact of LDs, an additional set of simulations was performed evaluating a 3 g LD—administered in the same manner—with maintenance regimen unchanged, in both ECMO and non-ECMO patients. For each regimen, 1,000 simulated profiles were generated using semi-parametric sampling methodology using the final model (29). Predictions were generated every 0.2 h for the first 24 h of the dosing interval. Results of the CFR analysis were visualized using the ggplot package for R (31).

## RESULTS

### Patient characteristics

A total of 70 patients (60% male) provided 114 plasma samples for analysis (1–14 per patient). Table 1 summarizes the demographic and clinical characteristics of these patients. Mean ± SD age was 62.1 ± 14.4 years, the mean ± SD wt was 83.3 ± 26.5 kg, and the mean ± SD BSA was 1.96 ± 0.33 m^2^. The mean ± SD baseline CrCl was 115.8 ± 88.6 mL/min. The mean ± SD initial 24 h dose was 4.2 ± 1.7 g per day. Measured plasma concentrations ranged from 1.7 to 142.9 mg/L, and the mean ± SD time after dose for observed samples was 7.8 ± 6 h. Figure S2 shows the observed cefepime concentrations relative to the time after the last dose.

**TABLE 1:** Baseline demographics and clinical characteristics^*a*^

Demographics (*N* = 70)	Measurement
Age (years)^*b*^	62.1 ± 14.4
TBW (kg)^*b*^	83.3 ± 26.5
BSA (m*^2^*)^*b*^	1.96 ± 0.33
SCr (mg/dL)^*b*^	1.12 ± 0.66
CrCl (mL/min)^*b*^	115.8 ± 88.6
ECMO *n* (%)	9 (12.9%)
Samples per patient	
ECMO^*c*^	2 [1–6]
Non-ECMO^*c*^	2 [1–3]
Sex *n* (%)	
Male	42 (60%)
Female	28 (40%)

^*a*^ Body surface area (BSA), Cockcroft-Gault estimated creatinine clearance (CrCl), extracorporeal membrane oxygenation (ECMO), serum creatinine (SCr), Total body weight (TBW).

^*b*^ Mean ± SD.

^*c*^ Median [IQR].

^*d*^ Empty cells differentiate the measures of n(%) vs mean+/-SD or median IQR.

**Fig 1:**
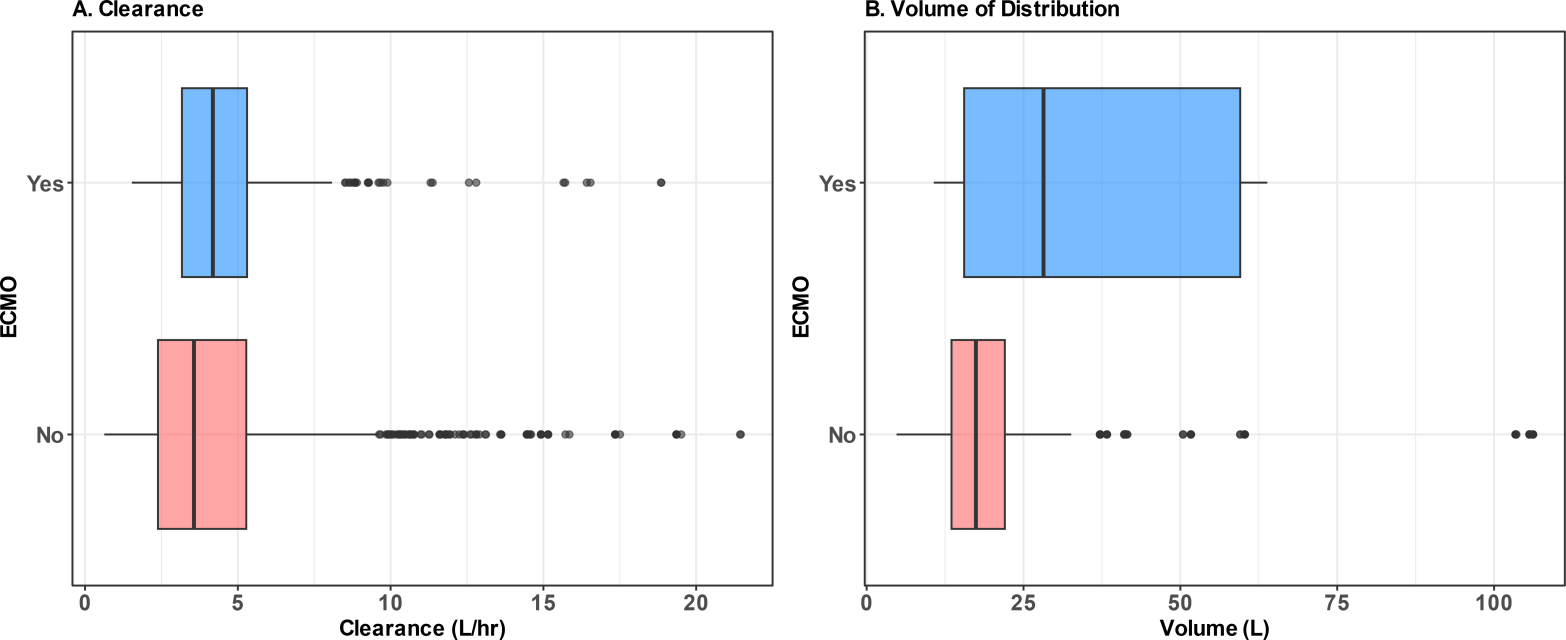
Effect of ECMO on cefepime clearance (**A**) and volume of distribution (**B**). (**A**) CL and (**B**) Vd stratified by ECMO status. Boxplots display the median, IQR, and outlier values for the Bayesian posterior distribution based on covariate values across all time points stratified by ECMO status. Results reported in text are the Bayesian posterior point estimates for each patient based on covariate values at time zero stratified by ECMO status.

ECMO was required in 13% (*n* = 9) of patients during treatment. The median number of samples collected per patient was similar between ECMO and non-ECMO groups (two samples per patient, respectively). Figure 1 displays the relationship between ECMO on PK parameters in the unadjusted PK model. The median (interquartile range [IQR]) CL for patients on ECMO was 4.6 (3.6–6.7) L/h, which was greater than the median CL of patients not receiving ECMO (3.12 [2.0–4.1] L/h). The median Vd for patients on ECMO was 23 L (IQR: 15.4–50.4 L), which was greater than the median Vd of those not receiving ECMO (17.1 [2.9–23.1] L). The inclusion of ECMO in the population reduced the overall OFV by >3.84 (*P* < 0.05), as described in the model build below and Table S1.

### Model development and selection

A two-compartment model (−2 * LL = 947.3) provided a significantly better fit of the observed data vs a one-compartment model (−2 * LL = 957.3; ΔOFV = 10) and thus served as the base model for subsequent comparisons. Table S1 provides a detailed comparison of the model build. Covariate models integrating renal (i.e., CrCl on CL [ΔOFV = 41.8]) and non-renal effects on CL (ΔOFV = 5) as well as total body weight effects on Vd (ΔOFV = 8.9) yielded significant improvements in fitness. Non-linear scaling of CrCl on CL yielded further improvements (ΔOFV = 6.6). Models evaluating the effect of ECMO on CL (ΔOFV = 10.2) and Vd (ΔOFV = 8.7) each yielded improvements in model performance individually but not when combined (Table S1). The model parameterized with an effect of ECMO on CL was rejected due to low precision (i.e., CV > 200%) and point estimates near zero (i.e., a null effect). After backward elimination, the non-renal CL term was removed (ΔOFV = −3.6), after which no other covariates qualified for elimination (Table S1). Covariate equations for the final model are shown in equations 1 and 2:


(1) $$CL=CL1\times {\left(\frac{CrCL}{120}\right)}^{\beta 1}$$

(2) $$Vd=V1\times \frac{WT}{70}\times e^{\beta 2\times ECMO}$$

The term *CL1* is scaled total CL to CrCl standardized to 120 mL/min. The term β1 is a non-linear scaling term modifying the relationship between CrCl and CL. The term *V1* scaled central Vd to body weight (WT) standardized to 70 kg. The term β2 is the proportional change in Vd for a patient requiring ECMO compared to a non-ECMO patient. The term β2 represents the proportional change in Vd associated with ECMO cannulation, where ECMO is treated as a binary classifier (0 = no ECMO, 1 = ECMO). This formulation allows β2 to quantify the population increase in Vd for ECMO patients relative to non-ECMO patients. Table 2 provides a summary of the population PK parameters.

**TABLE 2:** Median parameter values and 95% credible intervals from the final population PK model^*a*^

Parameter	Median (weighted)	95% Credible interval
CL1 (L/h):	4.45	3.22–5.37
β1	0.76	0.52–0.92
V1 (L):	16.3	6.54–33.5
β2	1.043	−0.40–1.59
KCP (h^−1^):	3.23	0.49–8.61
KPC (h^−1^):	2.68	2.01–9.12

^*a*^ Median (weighted) population PK parameters and associated 95% credible intervals for cefepime in patients with and without ECMO. Parameters include population typical values for creatinine clearance normalized clearance (CL1 in L/h), non-linear scaling effect of clearance [i.e., (CrCl/120 mL/min)^β1^], weight-normalized (WT/70 kg) volume of distribution (V1 in L), the proportional change in Vd for ECMO patients (β2 * ECMO), and intercompartmental transfer rates (KPC and KCP in h^-1^).

**Fig 2:**
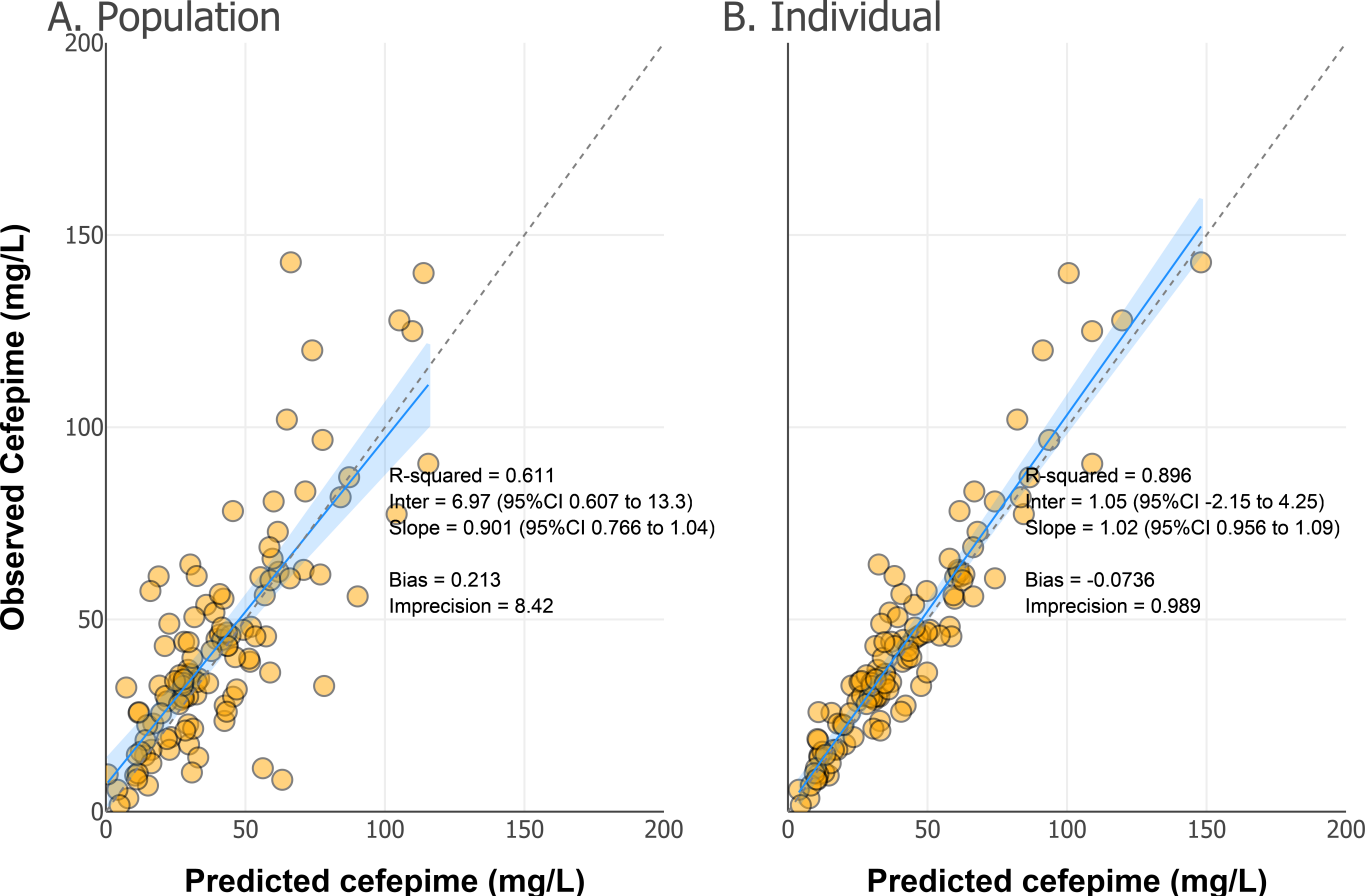
Population (**A**) and individual-predicted concentrations (**B**) versus observed concentrations from the final covariate-adjusted PK model. Orange circles represent individual data points, and the solid line indicates the line of unity (perfect prediction). The shaded blue area denotes the 95% CI for the model’s predictive performance. Goodness-of-fit statistics for the population (**A**) and individual (**B**) predictions included *R*^2^, slope, and intercept of regression lines as well as bias and imprecision.

The population and individual observed versus predicted concentrations for the final model are shown in Fig. 2. The population model predictions yielded an *R*^2^ of 0.611, a slope of 0.901 (95% CI: 0.766 to 1.04), and an intercept of 6.97 (95% CI: 0.607 to 13.3). The final population model had low bias (0.213 mg/L) and imprecision (8.42 mg^2^/L^2^). The posterior predictions yielded an *R*^2^ of 0.896, a slope of 1.02 (95% CI: 0.956 to 1.09), and an intercept of 1.05 (95% CI: −2.15 to 4.25). The final individual model had low bias (−0.0736 mg/L) and imprecision (0.989 mg^2^/L^2^).

### Individual PK/PD target attainment

**Fig 3:**
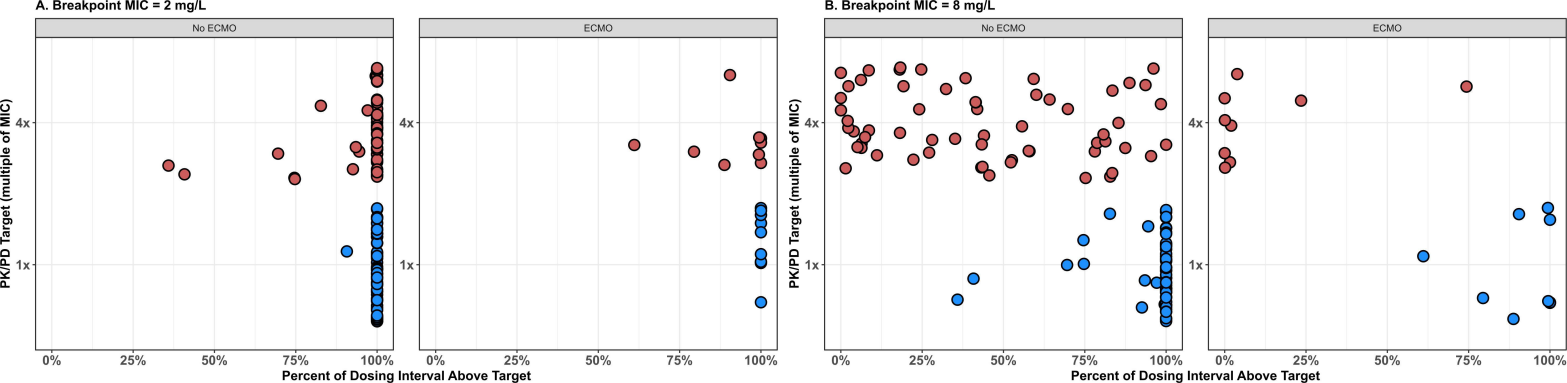
Individual target attainment vs (**A**) Enterobacterales and (B) *P. aeruginosa* breakpoints. Individual target attainment rates versus the cefepime (**A**) Enterobacterales (MIC = 2 mg/L) and (**B**) *P. aeruginosa* (MIC = 8 mg/L) breakpoint MICs. Red points evaluate attainment vs a more aggressive PK/PD target (100% *f*T_>4xMIC_), whereas blue points evaluate attainment vs a less aggressive PK/PD target (100% *f*T_>1xMIC_). Analyses are stratified by ECMO status.

Individual target attainment rates for the Enterobacterales breakpoint (MIC 2 mg/L) are shown in Fig. 3A stratified by ECMO status. At the 1× target for Enterobacterales (blue circles), both non-ECMO and ECMO patients demonstrated high rates of attainment (98.4%, *n* = 60/61 vs 100% *n* = 9/9). At the 4× target for Enterobacterales (red circles), non-ECMO patients had greater target attainment compared to ECMO patients (82%, *n* = 50/61 vs 55.6% *n* = 5/9). Individual target attainment rates for the *P. aeruginosa* breakpoint (MIC 8 mg/L) are shown in Fig. 3B. At the 1× target for *P. aeruginosa* (blue circles), results mirrored the Enterobacterales 4× MIC analysis (i.e., 2 mg/L × 4 = 8 mg/L). At the 4× target for *P. aeruginosa* (red circles), nearly all non-ECMO patients (98.3%) and all ECMO patients (100%) did not reach 100% *f*T_>4xMIC_.

### Monte Carlo simulations

**Fig 4:**
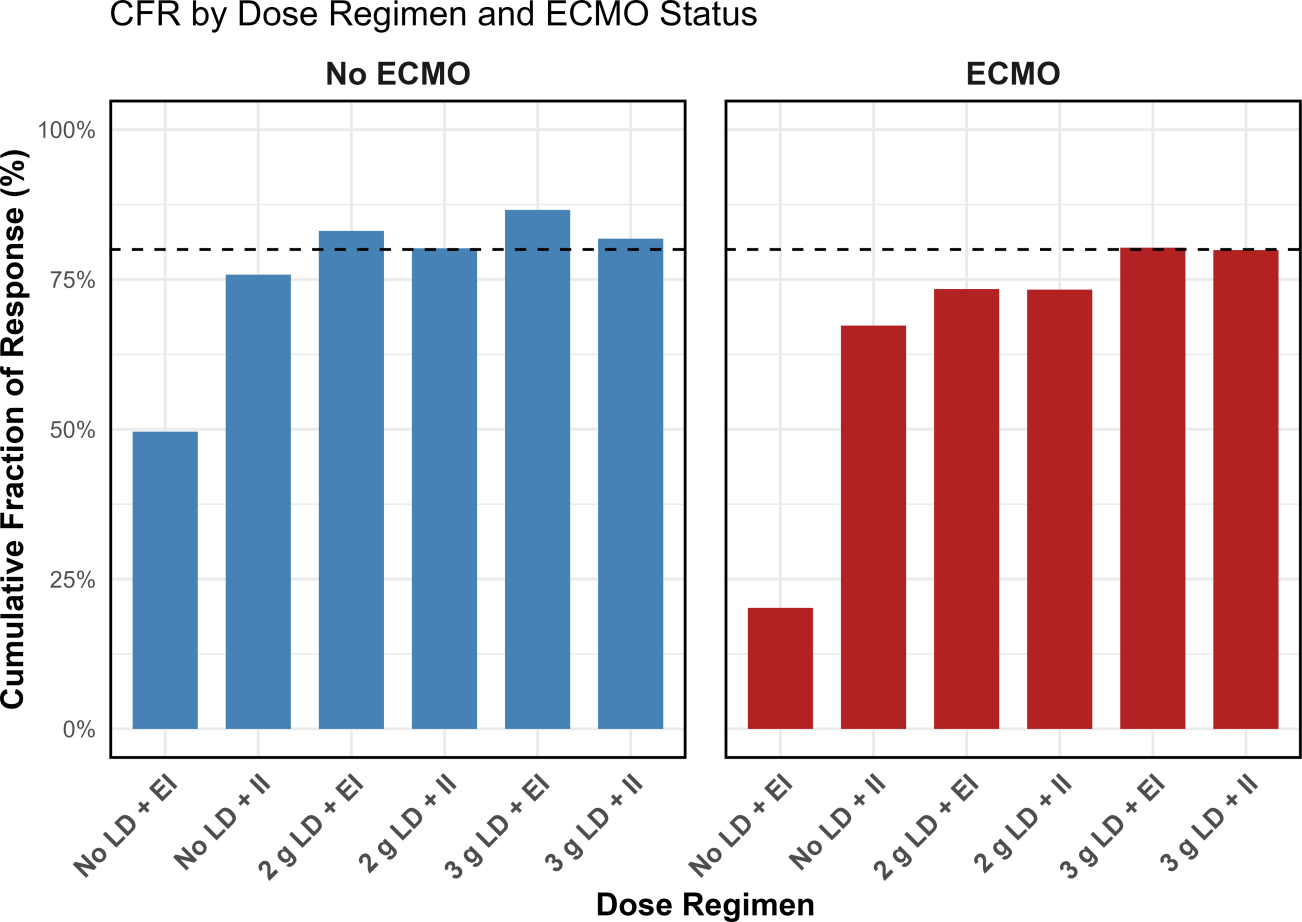
Simulated cefepime target attainment vs the EUCAST MIC distribution of *P. aeruginosa* with and without loading doses employed. CFR for cefepime at 100% *f*T_>1xMIC_ stratified by ECMO status and dosing regimens. Blue bars represent non-ECMO patients, and red bars represent ECMO patients. The dashed line indicates CFR of 80%. Abbreviations: extended infusion (EI) of 2 g IV every 8 h, intermittent infusion (II) of 2 g IV every 8 h, No LD = no loading dose given.

Population-based simulations revealed that PTAs at a fixed MIC of 2 mg/L (i.e., the Enterobacterales breakpoint) were >97% for all regimens. However, at a fixed MIC of 8 mg/L (i.e., the *P. aeruginosa* breakpoint), ECMO patients receiving EI or II regimens without an LD achieved PTAs of 83.5% and 82.5%, respectively, which was below a target of 90%. Considering the full EUCAST MIC distribution for *P. aeruginosa* as a worst-case scenario, we found that CFR varied by ECMO status and the presence of LDs (Fig. 4).

Across both groups, adding an LD improved the CFR compared to non-LD (Fig. 4). In non-ECMO patients, no-LD regimens yielded CFRs of 49.4% (EI) and 75.7% (II), whereas a 2 g LD increased these to 83.1% and 80.2%, and a 3 g LD further boosted them to 86.5% and 81.8%. In ECMO patients, no-LD regimens produced much lower CFRs (20.2% for EI and 67.3% for II), and although a 2 g LD improved CFRs to 73.7% and 73.3%, only the 3 g LD regimens reached the 80% target (80.2% and 80.3%).

## DISCUSSION

Our population modeling approach identified a significant effect of ECMO on cefepime Vd. This may be due to an increased circulating plasma volume as a function of the extracorporeal circuit. Additionally, the severity of illness, including systemic inflammation and altered fluid balance, may further contribute to the observed increase in Vd (32). Integration of ECMO effects on Vd improved overall model fitness, and this effect was retained in our final population PK model according to improvements in −2 * LL, AIC, and biologic plausibility, which was considered fit for purpose. Using Bayesian estimates from our final PK model, we evaluated the effect of ECMO on the individual probability of target attainment rates. We found that ECMO patients were less likely to achieve 100% *f*T_>MIC_ against aggressive targets for Enterobacterales compared to non-ECMO patients. Likewise, we found that 100% *f*T_>MIC_ was more difficult for ECMO patients to achieve against *P. aeruginosa* as compared to non-ECMO patients. Nearly all patients failed to achieve 4× MIC targets against the *P. aeruginosa* breakpoint MIC.

The first 24 h of cefepime treatment were chosen as the focus of this analysis, and based on a calculated cefepime half-life of approximately 2.54 h, most patients would be expected to reach steady state within approximately 13 h of therapy initiation. This analysis captures both the initial distribution phase and an approximation of steady-state exposures for many patients. While the addition of loading doses improved target attainment during this timeframe, further optimization through therapeutic drug monitoring (TDM) may be necessary to maintain appropriate exposure beyond 24 h. Real-time TDM, particularly bedside or immediate feedback assays, could facilitate early dose adjustments and improve patient outcomes.

We also evaluated the population target attainment using Monte Carlo simulations to evaluate 100% *f*T_>MIC_ attainment rates against the Enterobacterales and *P. aeruginosa* breakpoint MICs. We found that PTA was acceptable against the breakpoint of 2 mg/L for all regimens. We also found that ECMO patients had sub-optimal PTA in the absence of an LD.

We also evaluated CFR versus the EUCAST MIC distribution for *P. aeruginosa*. We found that ECMO patients consistently had lower CFR compared to non-ECMO patients, even when LDs were administered. Our findings are likely driven by the median 2.8-fold greater Vd in ECMO patients as estimated by our final population model, leading to systematically lower predicted plasma levels. Given the relationship between increased Vd and cefepime distribution, we explored whether increasing the LD from 2 g to 3 g would improve CFR. In additional simulations, a 3 g LD led to improvements in CFR (Fig. 4). While increasing the loading dose may improve early target attainment, it is important to consider the potential for cefepime neurotoxicity, particularly in patients with impaired renal function or other risk factors (33). Close monitoring and individualized dosing, potentially guided by therapeutic drug monitoring, may be necessary to optimize efficacy while minimizing toxicity. Our CFR analysis using the EUCAST MIC distribution for *P. aeruginosa* represents a worst-case scenario, with CFR outcomes likely influenced by local resistance patterns. Cefepime dose individualization and early empiric combination treatment, until susceptibilities are known, may be required for ECMO patients given the risk of inadequate exposures in this population.

Prior studies have also found variable PK and PK/PD attainment among patients requiring ECMO. Zheng et al. evaluated cefepime concentrations in a group of matched (1:1) patients with and without ECMO requirements (12). Among cases and controls, CRRT was used in about 20% of patients, and CrCl was similar between groups. They found that cefepime peak, trough/MIC, and trough/MIC > 4 attainment rates were lower in ECMO patients. However, the authors did not identify a difference in cefepime elimination rate constant or Vd between the groups (12). A strength of the current study is that we included patients who were not on renal replacement, thus mitigating confounding due to RRT. Additional population PK studies incorporating ECMO and CRRT are needed. Another strength of our study was the use of a population modeling approach rather than a reliance on univariate associations to classify the effect of concurrent ECMO. In a separate study, Cheng et al. enrolled six patients requiring ECMO, one of whom required RRT, and conducted rich sampling and population PK analysis and simulations (10). In their cohort, the estimated population mean ± SD Cl and Vd were 2.43 ± 1.55 L/h and 15.1 ± 3.33 L, respectively. The median CrCl among ECMO patients in their cohort was 75 (IQR: 64–84) mL/min. In contrast, the mean ± SD baseline CrCl of patients in our study was 115.8 ± 88.6 mL/min, and the median population parameter values for CL and Vd in non-ECMO patients were 4.45 L/h and 16.3 L where ECMO patients had a 2.8-fold-greater Vd (i.e., 46.25 L vs 16.3 L). Kois et al. enrolled six ECMO patients (two on RRT) in a PK study and estimated population mean ± SD parameters for CL and Vd of 5.99 ± 2.03 L/h and 10.1 ± 4.88 L, respectively (11). In this study, CrCl was >100 mL/min among the non-RRT patients, likely explaining the higher estimated CL compared to Cheng et al. (10). One potential explanation for the larger Vd in our ECMO cohort compared to these prior studies is the physiological changes associated with veno-venous (VV) ECMO support. VV ECMO is commonly used for respiratory support in clinically ill patients and may lead to altered fluid balance, expansion of extracellular fluid volume, and other physiological changes that could contribute to an increased Vd. However, without detailed hemodynamic data or serum albumin levels, the precise mechanisms underlying the observed differences in Vd remain unclear. The timing of ECMO initiation could also play a role in influencing the PK, as this was not standardized across patients. Further studies are needed to explore the effects of ECMO and critical illness on drug distribution, as well as potential confounding factors. Thus, variation in cefepime PK parameters and secondary exposures in ECMO patients appears to be driven by a combination of factors, including critical illness factors, changes in renal function, and possibly other unmeasured physiological alterations.

The mechanism underlying the increased cefepime Vd in ECMO is unclear but is likely influenced by multiple factors, including the presence of several liters of circulating fluid outside the body, which serves as an additional compartment for water-soluble drugs. Our results suggest a higher Vd for cefepime in the setting of ECMO, a finding consistent with the expanded extracorporeal fluid volume associated with ECMO. *Ex vivo* studies of cefepime concentrations in ECMO circuitry have demonstrated that cefepime is not significantly sequestered in the circuit (8, 9). Destache et al. evaluated the pharmacokinetics of cefepime in ECMO patients and found no significant difference in cefepime clearance intra-ECMO vs pre-ECMO clinical sampling; however, they observed a greater cefepime area under the concentration time curve (AUC) intra-ECMO, potentially driven by drug accumulation with multiple dosing or circuit-induced alterations (34). We hypothesize that this expanded circulating blood volume, in addition to a greater severity of illness, plays a major role in the increased Vd observed in the ECMO patients. The association between greater illness severity and increased Vd may also be partially explained by endothelial dysfunction and capillary leak syndrome, both of which are common in critically ill patients and may contribute to altered drug distribution. Several aspects of our study likely allowed us to identify an effect of ECMO on Vd: first, the removal of CRRT patients from consideration in model development, and second, our population PK modeling approach, which explicitly incorporated ECMO as a covariate on Vd. This differs from prior studies that did not model ECMO effects directly or relied on smaller sample sizes, potentially limiting the ability to identify covariate relationships. Future studies should focus on correlating markers of illness severity, such as clinical scores or inflammatory markers, with cefepime PK parameters to better understand these relationships.

Our study has strengths and limitations. A notable strength was in our design: by focusing exclusively on patients not on CRRT or HD, we eliminated a major confounder in the analysis of cefepime clearance. This makes our findings particularly relevant for the initial phase of ECMO support, where the clinical picture is still evolving, and RRT therapies may not yet influence drug dosing. Another strength was our population PK approach: we leveraged data from 70 real-world adult patients prospectively enrolled in the SCRIPT study, thereby increasing generalizability. One limitation of our study is that this was a single-center cohort with nine ECMO patients. However, our study is the single largest population PK study of ECMO patients conducted to date. Another limitation is that we were unable to evaluate links between PK/PD targets and clinical outcomes, such as survival or infection resolution. However, these data are being collected in an ongoing manner. Additionally, the timing of ECMO initiation may introduce variability that could influence outcomes. Future studies are needed to confirm these findings and to explore the relationship between clinical outcomes, PK/PD attainment, and drug levels at the site of infection, such as the epithelial lining fluid, in critically ill patients with suspected pneumonia. Additionally, the absence of serum albumin data limits our ability to fully explain the increased Vd, and future studies should assess albumin’s role in drug distribution.

### Conclusion

We observed that ECMO was associated with a 2.8-fold increase in cefepime volume of distribution, suggesting a potential for increased extracellular fluid expansion in this population. Simulations revealed lower PK/PD target attainment among ECMO patients and that a 2 g loading dose was insufficient to overcome these alterations, leading to suboptimal target attainment, whereas a 3 g loading dose restored the cumulative fraction of response to at least 80% for both extended and intermittent infusion regimens.

## SUPPLEMENTAL MATERIAL

Fig. S1 aac.00102-25-s0001.pdf EUCAST MIC distribution for *P. aeruginosa* used in Monte Carlo simulations for CFR analyses.

Fig. S2 aac.00102-25-s0002.pdf Spaghetti plot of observed cefepime plasma concentrations in n=70 adult patients.

Table S1 aac.00102-25-s0003.pdf Covariate model evaluation in comparison to cefepime base models.

ASM does not own the copyrights to Supplemental Material that may be linked to, or accessed through, an article. The authors have granted ASM a non-exclusive, world-wide license to publish the Supplemental Material files. Please contact the corresponding author directly for reuse.
