# Supplementary material for: Individual target pharmacokinetic/pharmacodynamic attainment rates among cefepime-treated patients admitted to the ICU with hospital-acquired pneumonia with and without ECMO
Source: Antimicrob Agents Chemother. 2025 May 15;69(6):e00102-25. doi: 10.1128/aac.00102-25 (PMC12135513; doi:10.1128/aac.00102-25)

Figure S1. EUCAST MIC distribution for *P. aeruginosa* used in Monte Carlo simulations for CFR analyses

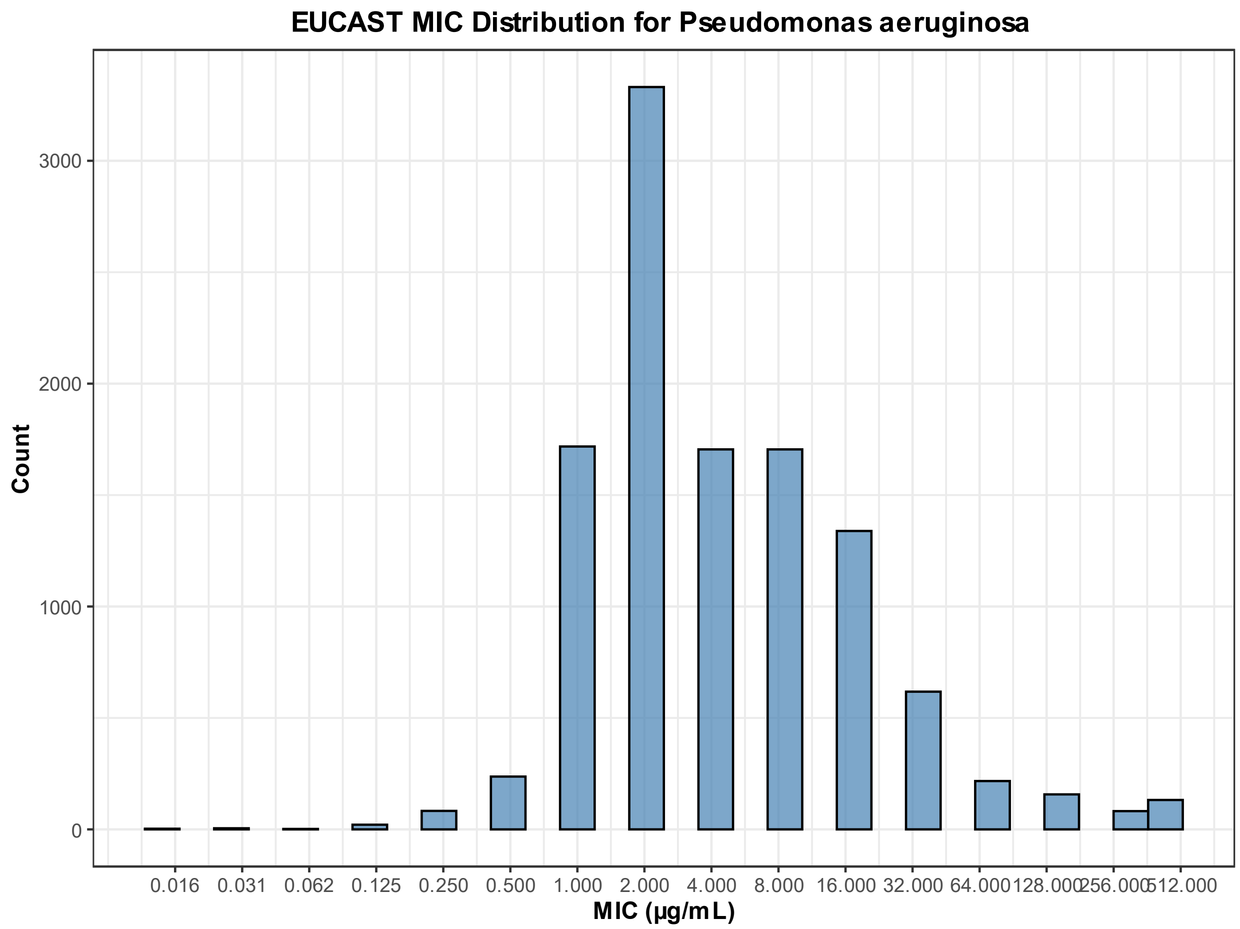

Supplement: Fig. S1 — EUCAST MIC distribution for P. aeruginosa used in Monte Carlo simulations for CFR analyses. [file aac.00102-25-s0001.pdf]
