# Supplementary material for: Individual target pharmacokinetic/pharmacodynamic attainment rates among cefepime-treated patients admitted to the ICU with hospital-acquired pneumonia with and without ECMO
Source: Antimicrob Agents Chemother. 2025 May 15;69(6):e00102-25. doi: 10.1128/aac.00102-25 (PMC12135513; doi:10.1128/aac.00102-25)
Supplement: Fig. S2 — Spaghetti plot of observed cefepime plasma concentrations in n=70 adult patients. [file aac.00102-25-s0002.pdf]

Figure S2. Spaghetti plot of observed cefepime plasma concentrations in n=70 adult patients

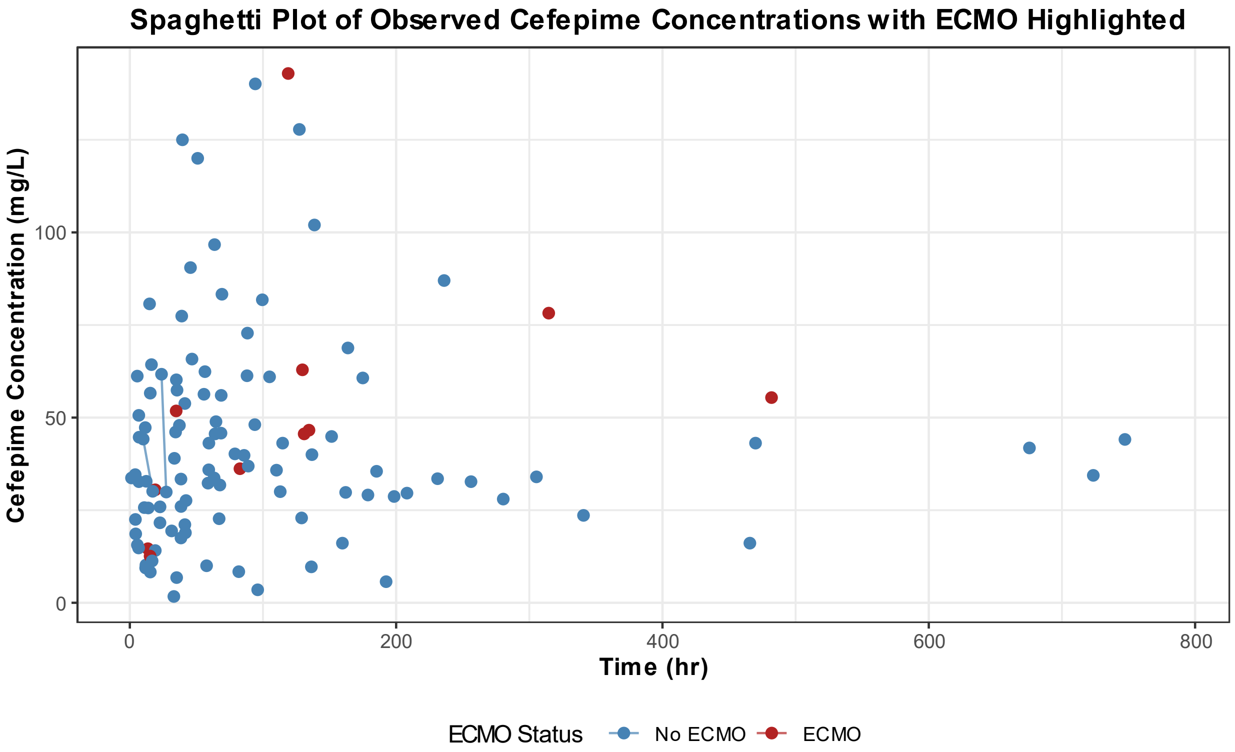

Figure legend: Lines represent concentrations from the same patient over time.
