# Supplementary material for: Individual target pharmacokinetic/pharmacodynamic attainment rates among cefepime-treated patients admitted to the ICU with hospital-acquired pneumonia with and without ECMO
Source: Antimicrob Agents Chemother. 2025 May 15;69(6):e00102-25. doi: 10.1128/aac.00102-25 (PMC12135513; doi:10.1128/aac.00102-25)
Supplement: Table S1 — Covariate model evaluation in comparison to cefepime base models. [file aac.00102-25-s0003.pdf]

Table S1: Covariate model evaluation in comparison to cefepime base models.

Run	*Model*	*Covariates*	*-2*LL*	*AIC*	*Comment*
1	1-compartment model: CL, V; Ke = CL/V	—	957.3	963.6	
2	2-compartment model: CL, V, KPC, KCP	—	947.3	957.9	Base model
3	Model 2 with CL scaled to (CrCl/120)	CrCl	905.5	916.1	
4	Model 3 with non-renal CL estimate	CrCl	900.5	913.3	intercept CL0
5	Model 4 with V scaled to WT/70	CrCl, WT	891.6	904.3	
6	Model 5 with β1 scaling renal CL	CrCl, WT	885.0	900.1	$(CrCL/120)^{\beta1}$
7	Model 6 with ECMO on CL: $CL1 * e^{(\beta2*ECMO)}$	CrCl, WT	874.8	892.2	β2 high CV%
8	Model 6 with ECMO on V: $V1 * e^{(\beta2*ECMO)}$	CrCl, WT	876.5	893.9	Lowest AIC
9	Combined model 7 and 8 ECMO effects	CrCl, WT	878.0	897.7	OFV > model 8
10	Elimination of CL0 from model 8	CrCl, WT	880.1	895.1	ΔOFV < 3.84; final
11	Elimination of β1 from model 10	CrCl, WT	885.6	898.4	ΔOFV > 3.84; stop

Table legend: The table outlines the progression of model-building steps, detailing the structural modifications, covariates incorporated, and their effect on model fit as assessed by -2*LL, and AIC. Decisions to accept or reject models were based on improvements in statistical metrics and parameter precisions, with the final model incorporating clearance and volume adjustments for renal function and ECMO. Parameters include population typical values for creatinine clearance normalized clearance (CL1 in L/hr), non-linear scaling effect of clearance [i.e., $(CrCl/120\ mL/min)^{\beta1}$], weight-normalized (WT/70 kg) volume of distribution (V1 in L), the proportional change in Vd for ECMO patients (β2 * ECMO), and intercompartmental transfer rates (KPC and KCP in hr^{-1}).
